# No Substrate Left behind—Mining of Shotgun Proteomics Datasets Rescues Evidence of Proteolysis by SARS-CoV-2 3CL^pro^ Main Protease

**DOI:** 10.3390/ijms24108723

**Published:** 2023-05-13

**Authors:** Peter A. Bell, Christopher M. Overall

**Affiliations:** 1 Department of Oral Biological and Medical Sciences, University of British Columbia, Vancouver, BC V6T 1Z3, Canada; peter.bell@ubc.ca; 2 Centre for Blood Research, Life Sciences Institute, University of British Columbia, Vancouver, BC V6T 1Z3, Canada; 3 Department of Biochemistry and Molecular Biology, University of British Columbia, Vancouver, BC V6T 1Z3, Canada

**Keywords:** protease, substrate, proteolysis, terminomics, TAILS, data mining, SARS-CoV-2, host–virus interaction, 3CL^pro^, Mpro, proteomics, MSFragger, FragPipe

## Abstract

Proteolytic processing is the most ubiquitous post-translational modification and regulator of protein function. To identify protease substrates, and hence the function of proteases, terminomics workflows have been developed to enrich and detect proteolytically generated protein termini from mass spectrometry data. The mining of shotgun proteomics datasets for such ‘neo’-termini, to increase the understanding of proteolytic processing, is an underutilized opportunity. However, to date, this approach has been hindered by the lack of software with sufficient speed to make searching for the relatively low numbers of protease-generated semi-tryptic peptides present in non-enriched samples viable. We reanalyzed published shotgun proteomics datasets for evidence of proteolytic processing in COVID-19 using the recently upgraded MSFragger/FragPipe software, which searches data with a speed that is an order of magnitude greater than many equivalent tools. The number of protein termini identified was higher than expected and constituted around half the number of termini detected by two different N-terminomics methods. We identified neo-N- and C-termini generated during SARS-CoV-2 infection that were indicative of proteolysis and were mediated by both viral and host proteases—a number of which had been recently validated by in vitro assays. Thus, re-analyzing existing shotgun proteomics data is a valuable adjunct for terminomics research that can be readily tapped (for example, in the next pandemic where data would be scarce) to increase the understanding of protease function and virus–host interactions, or other diverse biological processes.

## 1. Introduction

Protein termini can be classified as three main types: **original** (as translated from the open reading frame), **mature** (generated during protein maturation and cellular deployment), and **neo** (generated by the proteolytic processing of the parent protein). Proteolysis is a ubiquitous post-translational modification (PTM) that irreversibly alters protein function [1]. Substrate cleavage can have wide-ranging consequences: from the remodeling of protein complexes and the relocalization of cleavage products to the activation or abolition of enzymatic activity. System-wide detection of proteolysis is typically performed en masse via terminomics workflows that enrich protein terminal peptides in samples prior to analysis by mass spectrometry (MS). These methods exploit differences in the chemical properties of peptide N-termini, C-termini, and amino acid side chains for enrichment. Consequently, terminomics workflows, such as the terminal amine isotopic labeling of substrates (TAILS) [2], carboxy-TAILS (C-TAILS) [3], and the high-efficiency undecanal-based N-termini enrichment (HUNTER) [4], are specific for protein termini via their depletion of the internal peptides that are generated by trypsin or other proteases [5] in proteomic workflows during sample preparation. Tryptic peptides do not directly provide information on proteolysis, so their depletion targets MS analysis to the protease-generated semi-tryptic peptides.

Typical for dedicated PTM-specific workflows, terminomics is not routinely applied, and proteolysis remains under-investigated or is not even considered for most of the samples or experimental conditions reported. With the average number of tryptic peptides per mammalian protein that can theoretically be detected by MS ~20, most proteomic datasets will contain ~5% N- and ~5% C-terminal peptides. Thus, the sheer quantity of original, mature, and neo-terminal semi-tryptic peptides held in proteomics repositories means that they are a rich, untapped source of data on the experimental verification of original and mature termini vs. predictions and—for neo-termini—for proteolytic processing. The mining of non-enriched proteomics datasets for neo-termini has traditionally been hindered by two bottlenecks, which have prevented its widespread adoption in data analysis. Traditionally, a significant proportion of the time required for terminomics analysis is consumed by searching proteomics datasets. In this process, the mass information derived from MS is compared to a database containing the masses of all peptides that are theoretically present in a given sample. To detect the evidence of proteolytic processing (i.e., neo-termini), the database of possible peptide sequences must be expanded to include the potential truncation of N or C-termini. This ‘semi-specific’ search increases the size of the database exponentially, which significantly increases the computational processing time. Therefore, mining the unenriched datasets has typically been an inefficient way to detect proteolysis and has not been amenable to exploratory analyses. However, recent advances in MS search algorithms and analysis software (e.g., MSFragger [6]/FragPipe) now enable the searching of datasets to be 10–20× faster than previous algorithms [7]. These developments constitute a seismic change for terminomics research. In addition, the time associated with data analysis is no longer a significant limiting factor. In parallel, there have been rapid advances in MS instrumentation that can now acquire more high-resolution spectra per unit of time than was previously possible, and with greater sensitivity.

We hypothesized that these two innovations would enable the detection of high numbers of protein termini in non-enriched proteomics datasets. We tested this by comparing the number of protein termini that could be detected in non-enriched shotgun analyses vs. N-terminally enriched samples from two different laboratories. We found extensive evidence of proteolytic processing in the non-enriched samples, which validates this approach for some applications. Furthermore, our work led to the discovery of new substrates of the main SARS-CoV-2 3CL^pro^ protease, and revealed the cleavage of spike and other viral proteins during SARS-CoV-2 infection at previously unreported cleavage sites. Our findings demonstrate that the re-analysis of existing proteomics data is a valuable alternate strategy to complement dedicated broad-coverage terminomics approaches, which can be employed to increase our understanding of diverse biological processes.

## 2. Results

### 2.1. Non-Terminomics Proteomics Datasets Are an Abundant Source of Protein Termini for Terminomics Research

**Figure 1:**
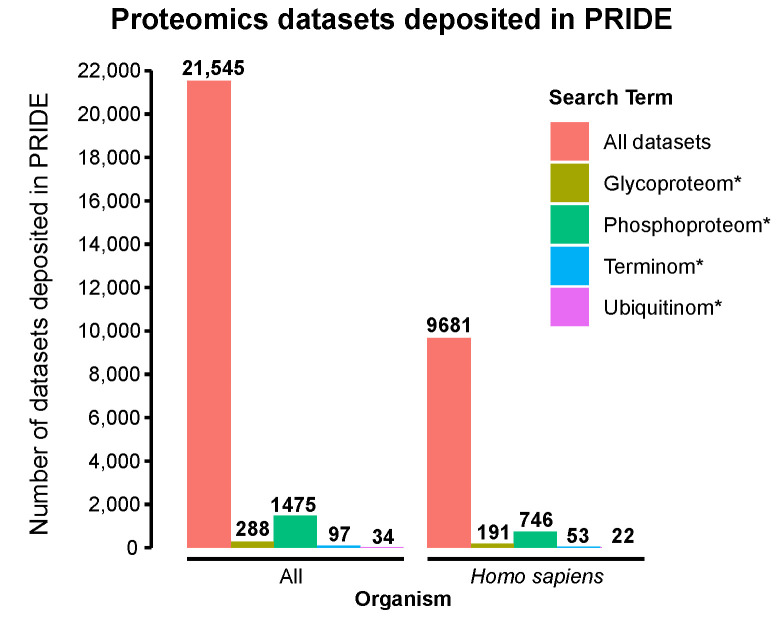
Terminomics workflows are not routinely applied in proteomics analyses. The bar charts show the results of keyword searches of the PRIDE proteomics database. The number of dataset entries containing search terms corresponding to different PTMs are shown for all organisms (**left**) or human datasets only (**right**). * signifies any combination of characters.

To estimate the number of proteomics datasets that are available for data mining, we performed keyword searches in the PRoteomics IDEntifications Database (PRIDE) Archive [8] (Figure 1). The number of terminomics datasets (defined as including the term ‘terminom*’, where * signifies any combination of characters) was low (N = 97) compared to the total number of deposited datasets (N = 21,545). Only 0.5% of the human-derived datasets (53 of 9681) met these criteria, which was 14-fold lower than the proportion of human datasets that were enriched for phosphorylation (7.7%). Thus, terminomics methods have not been applied to the majority of human sample types and experimental conditions. Therefore, data mining of shotgun proteomics datasets for neo-termini presents a new opportunity to increase our understanding of proteolytic processing in diverse biological processes, sample types, conditions, and stimuli.

### 2.2. Mining of Non-Enriched Datasets Yields High Confidence Substrates of SARS-CoV-2 3CL^pro^

**Figure 2:**
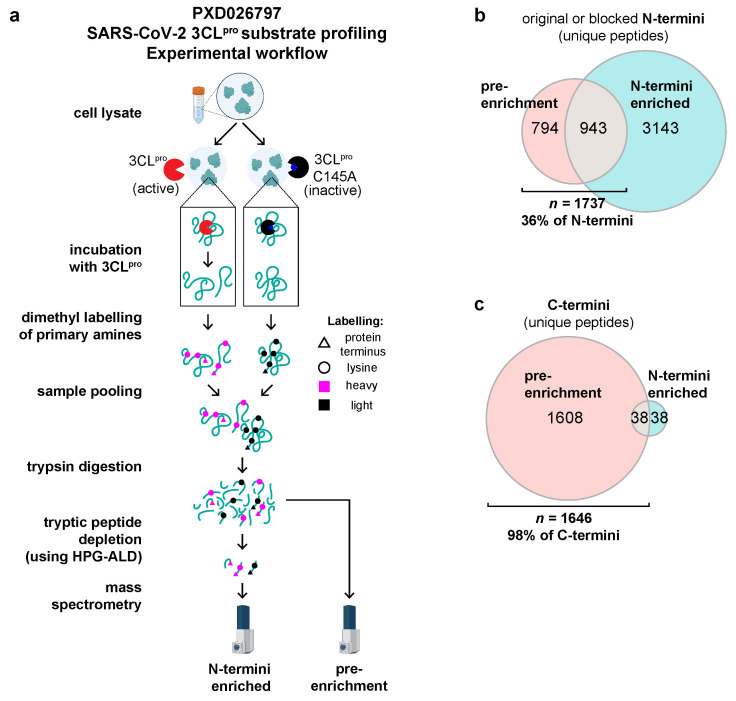
Non-enriched (shotgun) proteomics datasets are an abundant source of protein termini for terminomics research. (**a**) The TAILS experimental workflow used by Pablos et al. [9] for generation of the N-termini-enriched data and the pre-enrichment (preTAILS) shotgun data deposited in PRIDE entry PXD026797. HPG-ALD = hyperbranched polyglycerol-aldehyde. (**b**,**c**) The Venn diagrams that show the number of unique peptides with blocked N-termini (**b**) or unmodified C-termini (**c**) that were detected in the reanalysis of raw data from PXD026797. Note, in (N-)TAILS, no chemical labeling of the C-termini was performed, which requires a substantial modification of the TAILS, thus termed C-TAILS [3].

To compare the number of protein termini that can be detected in non-enriched samples versus samples that have been enriched for N-termini, we reanalyzed the raw MS data (PXD026797) from our TAILS terminomics investigation of the host cell substrates of the SARS-CoV-2 3CL^pro^ protease [9]. In this previous study, we incubated recombinant 3CL^pro^ or catalytically inactive mutant 3CL^pro^ C145A with cell lysates as the control; then, the N-termini were labeled by dimethylation at the protein level before trypsinization and N-termini enrichment by TAILS (Figure 2a). Notably, of the 6564 N-termini detected in all of the samples combined (comprising original, mature, naturally blocked, and experimentally labeled neo-N-termini), 36% (N = 1737) were detected in pre-enrichment (preTAILS or shotgun) samples (Figure 2b). Unsurprisingly, since C-termini are not targeted by (N)-TAILS, 95% of the C-termini detected by our analysis (N = 1608) were only found in pre-enrichment samples (Figure 2c). Combined, 3383 (52%) of the total N- and C-protein termini detected in the entire dataset were identified in pre-enrichment samples, and of this number, 1632 (48%) were neo-termini (see below and Supplementary Tables S1 and S2). These data demonstrate that significant numbers of protein termini can be detected in proteomics datasets, even in the absence of specific enrichment of samples for N- or C-termini.

**Figure 3:**
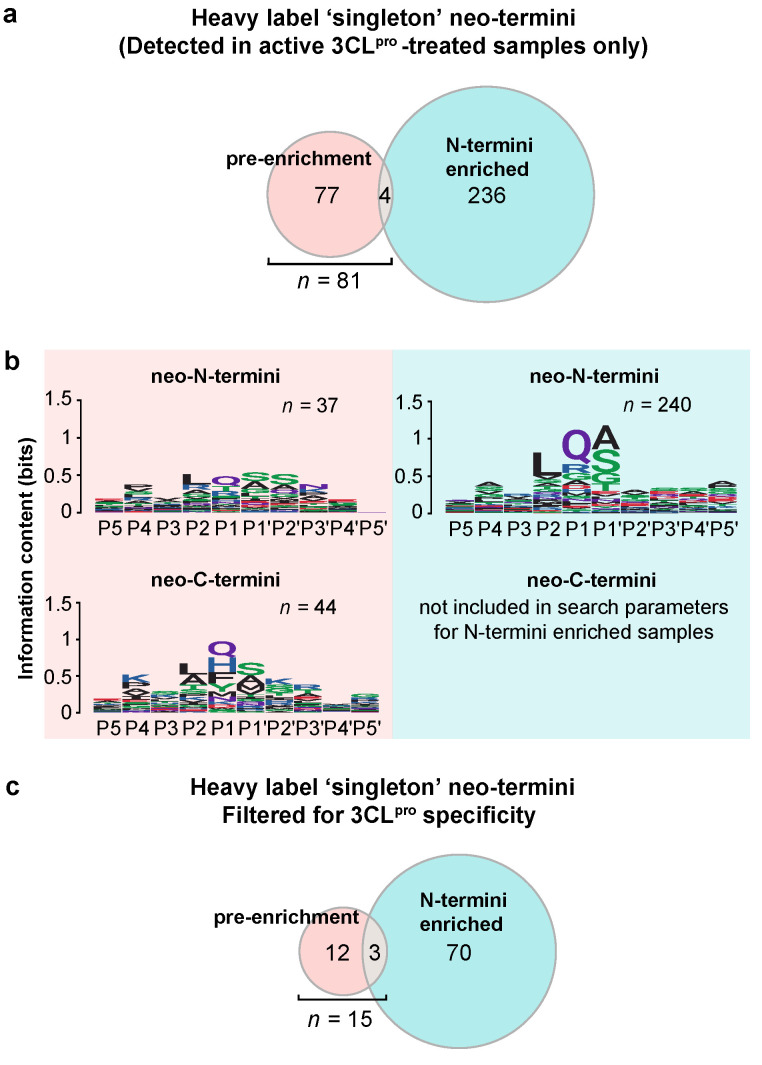
Reanalysis of the data of Pablos et al. [9] from non-enriched samples identified the C-terminal peptides of multiple SARS-CoV-2 3CL^pro^ substrate cleavage sites that were complementary to those detected in N-termini enriched samples: (**a**) The Venn diagram shows the number of neo-N- and C-termini that were only detected in the heavy-dimethyl-labeled (active 3CL^pro^-treated) samples (i.e., ‘singleton’ neo-termini); (**b**) the sequence logos of the singleton neo-N- and neo-C-termini were detected by reanalysis of the raw data from PXD026797; (**c**) the neo-termini from panel (**a**) were filtered based on known viral polyprotein 3CL^pro^ cleavage specificities. Note, Pablos et al. [9] reported that ~10% of substrate cleavage sites occurred with a ‘noncanonical’ P1 His or Met, but for these analyses these sites were not considered.

To probe the biological insights that can be derived from non-enriched samples, we compared the number of candidate 3CL^pro^ cleavage sites that were identified prior to, or following, N-termini enrichment. In our previous analysis [9] (which utilized Byonic (v.3.8.13) and Skyline (v.20.1.0.155) software for data processing), we used highly stringent winnowing criteria that only considered the neo-N-termini identified by a heavy label (i.e., detected only in the active 3CL^pro^-treated samples) as evidence of 3CL^pro^ substrate cleavage. Applying the same criteria to results from our new FragPipe analysis, we found a total of 81 neo-termini in pre-enrichment samples (*n* = 37 N-termini and *n* = 44 C-termini) compared with the 240 neo-N-termini in the TAILS-enriched samples (Figure 3a). Cleavage sites derived from protein termini showed an enrichment for the sequence LQ↓S/G/A (Figure 3b), which is consistent with the known substrate preferences of 3CL^pro^ in the SARS-CoV-2 polyprotein [10,11]. After filtering cleavage sites based on the polyprotein specificity of 3CL^pro^, 15 candidate 3CL^pro^ substrates were identified in pre-enrichment samples—18% of the total (Figure 3c).

Despite acting as an effective filtering step for the identification of high-confidence candidate 3CL^pro^ substrates, we hypothesized that the exclusion of the neo-termini detected with a quantified light label (i.e., detected in samples incubated with inactive 3CL^pro^) or a heavy label was overly conservative. As a consequence of the algorithms employed for MS1-based quantitation, it is possible for a peptide to be erroneously ‘quantified’ in a sample based on chromatographic noise. By discarding these peptides, valid protease substrates would be incorrectly filtered out, resulting in lower assay sensitivity. Conversely, the inclusion of the quantified neo-termini that were identified with log2-transformed ratios (active/inactive protease treated) close to 0 would also be undesirable as it would result in a high false-discovery rate for 3CL^pro^ substrates.

**Figure 4:**
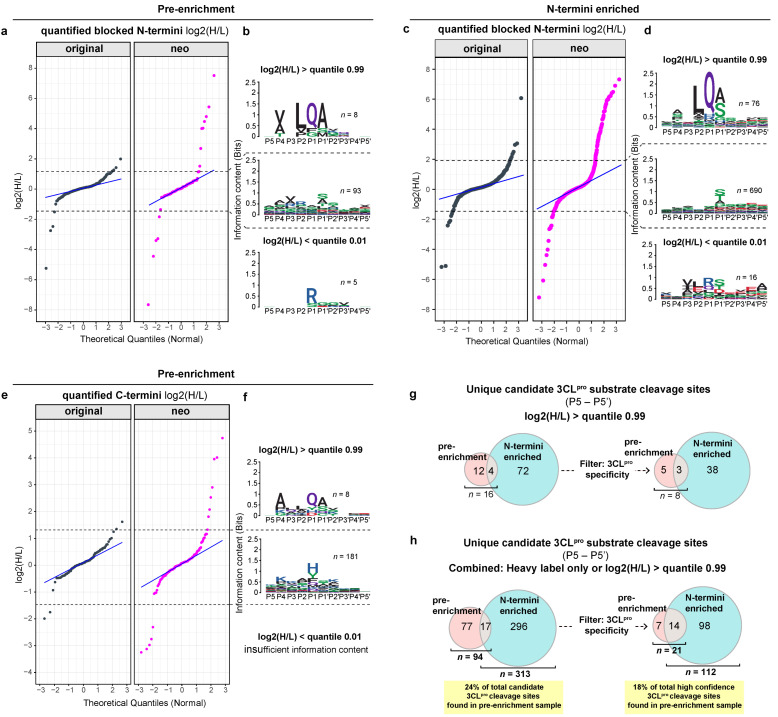
Original and mature protein termini inform fold-change thresholds for 3CL^pro^ substrate identification in the terminomics analyses of Pablos et al. [9]: (**a**,**c**,**e**) Quantile–quantile (Q-Q) plots show the log2(H/L) ratios of quantified protein N-termini (**a,c**) or C-termini (**e**), vs. normally distributed theoretical quantiles. The majority of original N- and C-termini followed a linear trend. A greater proportion of neo-N- (**a**) and C-termini (**e**) detected in pre-enrichment samples, or neo-N-termini detected in TAILS-N-termini-enriched samples (**c**) differed from normally distributed theoretical quantiles. Quantiles of 0.99 and 0.01 of the log2(H/L) ratios of original N-termini were used as upper and lower limits to classify the neo-termini that increased or decreased in abundance upon incubation with 3CL^pro^ (dotted line); (**b**–**f**) the sequence logos of cleavage sites corresponding to neo-N- (**b**,**d**) and C-termini (**f**) binned according to thresholds described above; (**g**) the Venn diagrams that compare the candidate 3CL^pro^ cleavage sites identified based on a log2(H/L) > quantile 0.99 of original peptides. Numbers are shown for pre-enrichment and N-termini enriched samples, with and without filtering for 3CL^pro^ SARS-CoV-2 polyprotein cleavage preferences; (**h**) the Venn diagrams compare all candidate 3CL^pro^ cleavage sites combined (‘singleton’ heavy label only and high log2(H/L) ratio). In total, 18% of all high-confidence 3CL^pro^ cleavage sites were found in pre-enrichment samples.

To address this sensitivity/precision trade off, we used the distribution of heavy/light (H/L)-labeled ion intensity ratios of the original N-termini as a ground truth upon which to base ratio cutoffs for neo-N- and C-termini (Figure 4). The ratios of the original N-termini are well-suited to this purpose because the probability of cleavage of any protein at its N-terminus (i.e., within the first ~25 amino acids) is very low given that only 742 3CL^pro^ cleavage sites have been reported within the entire human proteome [9,12,13,14]. Hence, the log2(H/L) ratio distribution of original N-termini reflects variability that is inherent to the assay and not to 3CL^pro^ activity. Quantile–quantile (Q-Q) plots were derived for quantified peptides, which showed that the log2(H/L) ratio of the majority of peptides followed a linear trend relative to the normally distributed theoretical quantiles (Figure 4a–e). Log2(H/L) ratios that corresponded to the quantiles of 0.99 and 0.01 of the original N-termini were used as upper and lower limits to classify the neo-termini that increased or decreased in abundance upon incubation and cleavage with 3CL^pro^. Cleavage sites derived from neo-termini > quantile of 0.99 showed a high similarity to the known viral polyprotein substrate specificity of 3CL^pro^ (Figure 4b,d,f). In contrast, the neo-termini < quantile of 0.01 were generally tryptic peptides (preceded by a P1 arginine only as the lysine is blocked in TAILS, see methods) that contained a consensus 3CL^pro^ cleavage sequence. Analysis based on log2(H/L) ratios resulted in the discovery of five 3CL^pro^ substrate cleavage sites that were unique to pre-enrichment samples (Figure 4g). Combined, 21 high-confidence 3CL^pro^ cleavage sites were identified in the pre-enrichment samples and formed 18% of the total (Figure 4h). Of these, 7 were unique to pre-enrichment samples (Table 1), and most were identified from the C-termini, which, as discussed, are not expected to be also present in the N-terminal enriched fraction. The remaining 14/21 high-confidence 3CL^pro^ cleavage sites were also identified in the N-termini enriched fraction.

These findings show that a significant proportion of the total proteolytic processing in a sample can be identified without enrichment for N- or C-termini. This demonstrates the utility of mining shotgun proteomics datasets for evidence of proteolysis and biological insights. Whilst the number of neo-termini that can be detected using this approach is lower than can be obtained by dedicated high-coverage terminomics workflows, this is still a significant proportion of the total and provides valuable launch points for further biological investigation by researchers not aiming to have full coverage analyses.

### 2.3. Identification of Viral Protein Proteolysis during SARS-CoV-2 Infection in Non-Enriched Datasets

**Figure 5:**
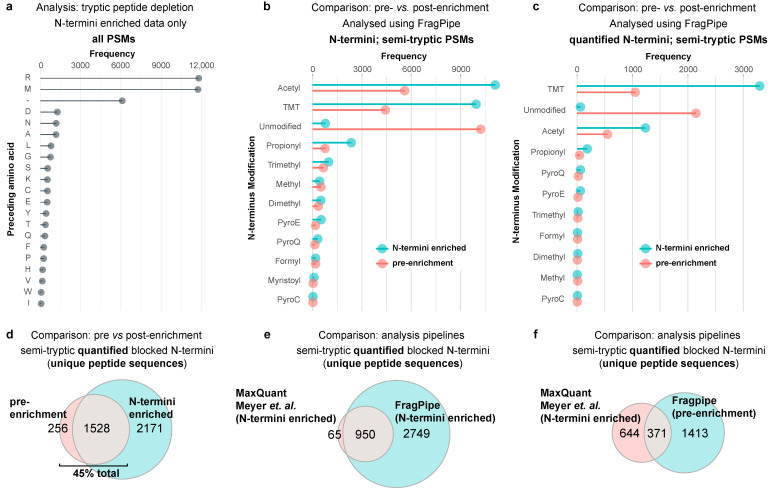
Re-analysis of the HUNTER-N-termini-enriched (PXD021152) and non-enriched (PXD021145) datasets of Myer et al. [13] from SARS-CoV-2-infected A549-ACE2 cells using FragPipe identifies additional protein termini. (**a**) Lollipop plot shows a high frequency of PSMs from N-termini-enriched samples with Arg as their preceding amino acid. These PSMs were considered ‘tryptic’ and excluded from subsequent analyses. (**b**,**c**) Lollipop plots show the frequency of different N-terminal modifications detected in our re-analysis of pre-enrichment and N-termini-enriched samples. (**b**) All PSMs. (**c**) Quantified PSMs only. (**d**–**f**) the Venn diagrams compare the unique quantified N-termini detected in (**d**) pre-enrichment vs. N-termini-enriched samples; (**e**); N-termini-enriched samples, as reported in Meyer et al. [13] vs. our reanalysis; and (**f**) N-termini-enriched samples (reported in Meyer et al. [13]) vs. pre-enrichment samples (our present analysis).

Next, we compared the biological insights that could be derived from the protein termini detected in non-enriched versus N-termini-enriched samples from the only terminomics analysis of SARS-CoV-2-infected human cells published to date [13] (Figure 5). The raw MS data from non-enriched (PXD021145) and N-termini enriched by HUNTER (PXD021152) samples were processed using FragPipe, and the number and positions of protein termini were compared. Whereas Pablos et al. [9] used isotopic-dimethylation to block the N-termini at the protein level, Meyer et al. [13] performed tandem mass tag (TMT)-labeling of protein N-termini. However, a FragPipe analysis of the N-termini-enriched HUNTER dataset revealed an incomplete depletion in the tryptic peptides inferred from the high frequency of peptide spectrum matches (PSMs) having arginine as their preceding amino acid in the P1 position of the cut site (Figure 5a). Consequently, N-terminally TMT-labeled peptides that were preceded in sequence by arginine were excluded from subsequent analyses. Unlike the Pablos et al. [9] dataset in which cell lysates were incubated with purified 3CL^pro^, the neo-termini isolated by Meyer et al. [13] were generated in infected living cells and were potentially subject to post-translational modification (PTM). Therefore, to maximize the detection of any modified N-termini and to determine the most relevant modifications to include for the mining of future datasets, we included common N-terminal PTMs [15] among the search parameters.

Strikingly, 5602 N-terminally acetylated PSMs were detected in the pre-enrichment sample (Figure 5b), of which 548 were quantified via a TMT labeling of lysine residues (Figure 5c). This compared favorably with the enriched sample, for which less than double the number of acetylated PSMs were detected (N = 11,113) despite enrichment for N-terminal peptides. A surprisingly high number of propionylated N-termini were also detected in both samples (pre-enrichment: N = 759; N-termini enriched: N = 2350). The functional significance of N-terminal propionylation relative to acetylation is not understood [16], but the high abundance of propionylated N-termini in this dataset shows that inclusion in search parameters is warranted for future terminomics data mining. A total of 4425 N-terminally TMT-labeled PSMs were detected in pre-enrichment fractions, of which 1050 were quantified (Figure 5c). Furthermore, 2145 unmodified semi-tryptic PSMs were quantified in the same sample. These peptides could have remained unmodified as a consequence of incomplete TMT-labeling of samples or else generated as an artifact by proteolysis following sample labeling. For this reason, only ‘blocked’ N-termini were included in subsequent analyses as these N-termini could only have been labeled if they were present in the original sample before TMT-labeling.

Of the total unique quantified blocked N-termini that were detected, 45% were present in the pre-enrichment sample (Figure 5d and Supplementary Tables S3 and S4). This further demonstrates the utility of non-enriched datasets for the identification of protein termini. The effectiveness of our FragPipe-based workflow was validated by comparison of the output of our analysis with the number of unique quantified N-termini that were reported by the same data by Meyer et al. [13] (Figure 5e). An additional 2749 peptides were detected by our analysis (i.e., an increase of 3.6-fold). The detection of quantified N-termini in pre-enrichment samples using FragPipe even compared favorably (a 1.8-fold increase) with the number of N-termini in the HUNTER-enriched samples that were analyzed by Meyer et al. using MaxQuant (v.1.6.7.0) (Figure 5f).

**Figure 6:**
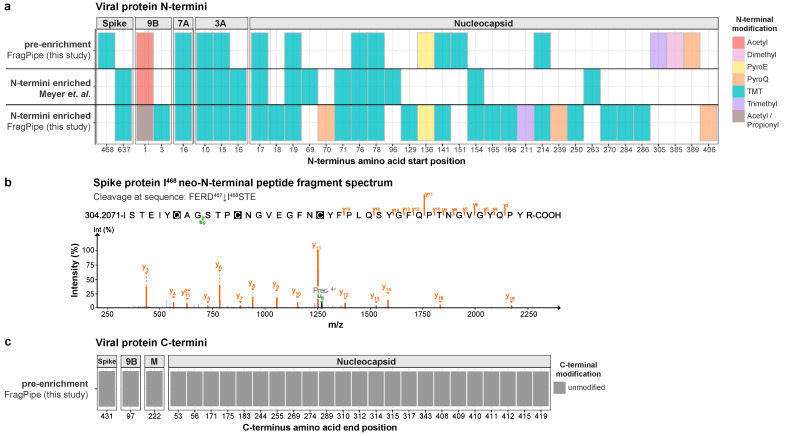
Identification of cleavage sites in SARS-CoV-2 proteins from non-enriched samples. (**a**) The amino acid positions of modified viral protein N-termini detected in our re-analysis of raw data from pre-enrichment (PXD021145) and N-termini-enriched (HUNTER) (PXD021152) samples from SARS-CoV-2-infected A549-ACE2 cells. The positions of N-termini reported by Meyer et al. [13] for the same N-termini-enriched dataset are also shown; (**b**) annotated fragmentation spectrum of neo-N-terminal peptide (amino acid position 468) derived from cleavage at FERD^467^↓I^468^STE from spike proteins as detected in pre-enrichment samples; (**c**) the amino acid positions of unmodified viral protein C-termini that were detected in our re-analysis of raw data from pre-enrichment samples.

Next, we compared the cleavage sites within viral proteins that were identified by our own analysis workflow via FragPipe with those detected via MaxQuant by Meyer et al. [13]. The analyses were broadly in agreement, with processing sites detected in common within Spike, 9B, 7A, 3A, and with nucleocapsid proteins (Figure 6a). Importantly, of the 15 neo-N-termini reported by Meyer et al. [13], we detected 8 in pre-enrichment fractions in addition to a further 8 cleavage sites, which were identified as various PTM-modified neo-N-termini (Figure 6a). These included a cleavage site (FERD^467^↓I^468^) within Spike (Figure 6b), which to our knowledge, has not previously been reported and has low homology to a caspase cleavage motif (DEVD) [17]. Analysis of the neo-C termini revealed evidence of cleavage of Spike at DFTG^431^↓C^432^ and of nucleocapsid at an additional 22 sites. In addition to the identification of the original C-termini of 9B viral protein, we also detected neo-C-termini in membrane protein and nucleocapsid (Figure 6c). Our findings demonstrate that the analysis of data derived from non-enriched samples can yield abundant evidence of viral protein cleavage during infection and, in so doing, shed light on host–virus interactions.

### 2.4. Identification of Infection-Induced Proteolysis in Non-Enriched Samples

**Figure 7:**
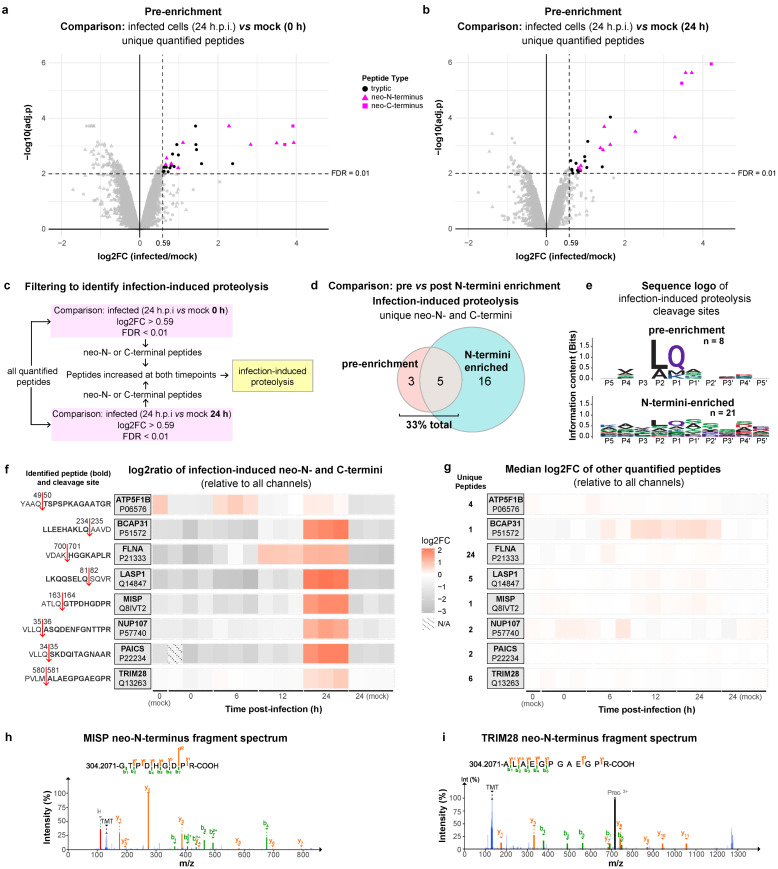
Identification of infection-induced proteolysis in non-enriched samples. (**a**,**b**) Volcano plots show peptides that were significantly increased in quantity in SARS-CoV-2-infected A549-ACE2 cells at 24 h.p.i. compared to mock controls at 0 h.p.i. (**a**), or 24 h.p.i. (**b**); (**c**) filtering strategy used to qualify the infection-induced neo-termini; (**d**) this Venn diagram compares the unique infection-induced neo-N- and C-termini detected in pre-enrichment vs. N-termini-enriched samples (HUNTER); (**e**) the sequence logos of infection-induced neo-termini; (**f**,**g**) these heatmap plots show the log2FC (fold change) of infection-induced neo-termini (**f**) or tryptic peptides from the same proteins (**g**) at 0, 6, 12, and 24 h.p.i. UniProt accession numbers are shown beneath the gene names for each protein. ↓, protease scissile bond. (**h**,**i**) Annotated fragmentation spectra of neo-N-terminal peptides from the MISP (**h**) and TRIM28 (**i**) detected in pre-enrichment samples.

Finally, we probed the pre-enrichment dataset of Meyer et al. [13] for evidence on the proteolytic processing of human proteins upon SARS-CoV-2 infection (Figure 7). The number of peptides identified in infected cells (24-h post-infection; h.p.i.) were compared with mock infection controls at 0 h (Figure 7a) and 24 h time points (Figure 7b). Limma-moderated t-statistics and Benjamini and Hochberg’s false discovery rate (FDR) adjustment were used to determine statistical significance. The neo-termini that were significantly increased at 24 h.p.i. versus both 0 h and 24 h mock controls were regarded as evidence of ‘infection-induced proteolysis’ (Figure 7c). There were eight such neo-termini detected in the pre-enrichment dataset (Figure 7d), of which five were also detected in the N-termini-enriched dataset. Sequence logo analysis of the infection-induced proteolysis cleavage sites identified in the pre-enrichment samples revealed a high similarity to the known substrate preferences of 3CL^pro^ (Figure 7e). Of the eight neo-termini, two were validated as 3CL^pro^ substrates (NUP107, cleavage at VLLQ^35^↓A^36^ and PAICS, cleavage at VLLQ^34^↓A^35^) and were detected in N-termini-enriched samples and validated by Meyer et al. [13]. Pablos et al. [9] also detected and validated the same cleavage site in NUP107. Four additional infection-induced proteolytic cleavage sites identified in BCAP31, LASP1, MISP, and TRIM28 (Figure 7f–i) were consistent with the sequence specificity of 3CL^pro^. In an independent investigation, we recently discovered that LASP1 and TRIM28 are bone fide substrates of 3CL^pro^ using orthogonal interactomics and biochemical cleavage assays [18]. BCAP31 and MISP are novel protease substrates in SARS-CoV-2 infection and here we identify them as high-confidence candidate 3CL^pro^ substrates for further validation. Furthermore, the cleavage of FLNA at VDAK^700^↓H^701^ by an unknown protease was induced at 12 and 24 h.p.i. To rule out the possibility that these neo-termini were significantly increased in ratio as a result of basal protein synthesis upregulation in infection, we analyzed the number of tryptic peptides from the same proteins upon infection (Figure 7g). A modest trend toward an increase in BCAP31 protein was inferred from a single tryptic peptide at 12 and 24 h.p.i. However, no significant increases in the overall expression of proteins containing infection-induced proteolysis cleavage sites were detected. Thus, our analysis further validated the 3CL^pro^ cleavage of NUP107 and PAICS during infection and identified four new high-confidence new candidate 3CL^pro^ substrates in a dataset that included samples that were not enriched for neo-termini, thus validating our approach.

## 3. Discussion

In this study, we show that proteomics datasets from non-enriched shotgun analysis of samples can be mined to detect significant numbers of protein termini using FragPipe v.19.1. Thus, the re-analysis of publicly available proteomics data is a valuable new complement to the terminomics toolbox. Sample enrichment for N- or C-termini prior to MS analysis remains the method of choice for the efficient high-coverage identification of cleavage sites; however, the mining of existing datasets presents an untapped opportunity for rapidly increasing our understanding of diverse biological processes and lowering the barrier of entry to terminomics research. Furthermore, it is the responsibility of the scientific community to minimize the environmental footprint of our research. By mining pre-existing datasets for new biological insights, we reduce the number of redundant investigations conducted, eliminate unnecessary waste, and increase research sustainability.

The ideal datasets to be analyzed using this approach will be those derived from the fractionated samples that have been acquired by modern MS instruments. This will increase the likelihood that low-abundance products of proteolysis will be detected. Furthermore, future meta-analyses that integrate multiple datasets from different experimental designs will increase confidence in the protein cleavage sites that are detected. Analysis of such a large quantity of data is computationally demanding. However, the speed of the tools integrated within FragPipe is already sufficient to feasibly scale up the mining of protein termini to hundreds of datasets—even without access to high-performance computing resources. Furthermore, FragPipe can be utilized to analyze datasets generated by all major MS vendors, using all labeled and label-free quantitation methods. This makes FragPipe an ideal platform for the high-throughput mining of protein termini.

Our analysis of non-enriched sample datasets affords an unexpected additional benefit in facilitating the detection of protein C-termini. Enrichment of samples for C-termini is technically challenging and is less commonly utilized compared to N-terminal enrichment methods [3]. Many protein cleavage sites are difficult to characterize from their neo-N-termini due to their sequence and the physiochemical properties of the peptides generated during sample preparation or the insufficient length for unequivocal identification. Thus, the identification of C-termini via data mining is a convenient and complementary means through which to increase the coverage of the human terminome. This point is exemplified by our identification of the infection-induced proteolysis of BCAP31 and LASP1 from the detection of neo-C-termini. The neo-N-terminus of the LASP1 generated upon cleavage at the same site would be intractable to detection by typical MS workflows due to an Arg residue in P4′, which would yield a peptide of sequence SQVR upon trypsin digestion. This peptide is too short to be unambiguously assigned to LASP1. Hence, identification of the LASP1 cleavage site by 3CL^pro^ in trypsin-digested samples is only possible by detecting the neo-C-terminus.

Consideration of the amino acid sequence surrounding protease cleavage sites highlights an important caveat for the mining of non-enriched datasets for protein termini. A common sample preparation step for negative enrichment N-terminomics workflows, such as TAILS and HUNTER, is the protein-level labeling of primary amines by dimethylation or TMT-labeling. In addition to blocking the protein N-termini, this also blocks the amine groups on Lys side chains, rendering the blocked peptides resistant to cleavage by trypsin at Lys, and consequently increases the average length of the peptides detected by N-terminomics. This is advantageous for terminomics. Were it not for the TMT blocking of Lys, then five of the eight infection-induced proteolysis sites that we identified in the Meyer et al. [13] dataset would not have been detected due to insufficient peptide length. TMT and dimethyl labeling are not performed prior to trypsin digestion during routine shotgun proteomics analysis as labeling after trypsinization increases the numbers of peptides that can be quantified. Therefore, there are differences in the neo-N- and C-termini that could be detected by the mining of standard shotgun proteomics datasets compared with the analysis of samples that have been Lys-blocked prior to trypsin digestion. Further, the identification of a blocked N-terminus unequivocally identifies such peptides as products of proteolysis in the biological sample.

Our analysis revealed new evidence of proteolysis of both host and viral proteins during SARS-CoV-2 infection and hence new candidate substrates. The functional effects of these cleavage events await further investigation. Even though we used the polyprotein cleavage site motif to guide the present analyses, it must be emphasized that noncanonical amino acids can quite often occur even for proteases with strict cleavage site specificities. For example, Pablos et al. [9] reported the occurrence of histidine or methionine in the P1 position of ~10% of 3CL^pro^ cleavage sites, which were noncanonical versus the canonical P1 glutamine. Thus, the numbers of cleavage sites reported here is an underestimation of the true number of substrates discovered for 3CL^pro^. Previously unreported cleavage sites in BCAP31, LASP1, MISP, and TRIM28 can likely be attributed to the activity of the viral protease 3CL^pro^, which has been implicated in the perturbation of numerous host processes [9,13,19,20]. The sequence of the VDAK^700^↓H^701^ cleavage site in FLNA makes it likely that infection-induced proteolysis is caused by the activity of a host protease. The consequences of an FLNA cleavage could be profound since the N-terminus of FLNA interacts with a variety of binding partners, including filamentous actin, TAF1B, MIS18BP1, and CRMP1 [7]. The removal of the FLNA N-terminus by proteolytic cleavage during SARS-CoV-2 infection may disrupt these interactions, resulting in a perturbation of the cytoskeleton. Indeed, multiple substrates of 3CL^pro^ within the Hippo pathway, which is involved in regulating and sensing cell shape changes, have been previously identified [9]. These findings, combined with our identification of proteolytic processing of viral proteins, demonstrate that re-analyzing existing proteomics data for neo-termini can yield new insights into host–virus interactions and other diverse biological processes. The stage is now set for the high-throughput mining of proteomics datasets and the expansion of the known human terminome.

## 4. Materials and Methods

### 4.1. Keyword Searches of PRIDE Archive

The PRIDE Archive [8] was accessed on 8 February 2023. Keyword searches of dataset entries were performed for different PTM-enriched dataset types using the following search terms: ‘glycoproteom*’ (glycoproteomics); ‘phosphoproteom*’ (phosphoproteomics); ‘terminom*’ (terminomics), and ‘ubiquitinom*’ (ubiquitinomics). (* signifies any combination of characters.)

### 4.2. Analysis of PXD026797 (Pablos et al. [9] Pre-Enrichment and TAILS N-Termini Enriched Datasets)

Raw data (.d files) were downloaded from PRIDE and converted to .mzml using msconvert v.3.0.22047-16f707e [21]. Data were then analyzed using FragPipe v.19.1 with the following tools installed: MSFragger v.19.1 [6], IonQuant v.1.8.10 [22], and Philosopher v.4.8.1 [23]. Proteomics data were searched against a database containing the UniProt Human Proteome (UP000005640), common contaminants, the SARS-CoV-2 proteome (UP000464024), and decoy sequences. Data from non-enriched (preTAILS) samples were searched with the following in silico digestion parameters: semi-specific ‘trypsin R’ digestion (i.e., cleavage C-terminal to Arg, not before Pro. Note, the lysine sidechain is also blocked by dimethylation and so is not susceptible to trypsin cleavage, which therefore cuts with trypsin R/ArgC specificity). In silico digestion parameters for N-termini enriched (TAILS) samples were set to semi-N-terminal-specific trypsin-R digestion. Search parameters common to all samples were as follows: one missed cleavage, and a precursor and fragment mass tolerance of ±20 ppm. The fixed modifications included in the searches were as follows: N-ethylmaleimide on Cys (+125.0477) and light dimethylation on Lys (+28.0313). The variable modifications included in searches were as follows: heavy dimethyl-labeling over light dimethylation on Lys (+6.0318); oxidation of Met (+15.9949); deamidation of Gln or Asn (+0.984016); acetylation of protein N-terminus (+42.0106); propionylation of protein N-terminus (+56.026215); peptide N-terminal light dimethylation (+28.0313); peptide N-terminal heavy dimethylation (+34.0631); peptide N-terminal pyroglu from Gln (−17.0265); and peptide N-terminal pyroglu from Glu (−18.0106). A false discovery rate (FDR) threshold of <0.01 was applied for the PSMs. An 8-core (16 Gb RAM) workstation was used for searches, which took between 5 h to complete for semi-N-terminal-specific searches, or 17 h to complete for semi-specific searches.

### 4.3. Filtering of Protein Termini Detected by Analysis of PXD026797

Where both heavy- (H) and light- (L) labeled forms of peptides were detected, the mean H/L ion intensity ratio for each peptide was calculated sequentially from technical and then biological replicates. The peptides meeting any one of the following criteria were filtered out prior to the Q-Q plot and sequence logo generation: (1) peptides identified by only a single PSM; (2) incorrect PSMs containing heavy-labeled lysine and light-labeled lysine modifications assigned to different lysine residues or N-termini within the same PSM; (3) peptides with pyro-Glu or pyro-Gln at the N-terminus preceded by Arg (rationale: cyclization can occur as an experimental artifact following trypsin digestion); and (4) N-terminal peptides with R as the previous amino acid and beginning with R (rationale: missed trypsin cleavages were responsible for the most highly variable (i.e., non-normal) H/L ratios among the original N-termini). The candidate 3CL^pro^ substrate cleavage sites were identified by heavy-labeled ‘singleton’ peptides or by log2(H/L) ratios > quantile of 0.99 of the original protein termini. The candidate 3CL^pro^ substrate cleavage sites were considered high confidence where the P2–P1 sequence matched the known 3CL^pro^ cleavage sites (L/F/V-Q↓A/S/N) within the SARS-CoV-2 polyprotein.

### 4.4. Analysis of Unenriched (PXD021145) and N-Termini Enriched (PXD021152) (Meyer et al. [13], Datasets)

The raw data (.raw files) were downloaded from PRIDE, converted to .mzml, and then analyzed using the software described in the section ‘Analysis of PXD026797’. Search parameters were as follows: N-term semi-specific ‘trypsin R’ digestion (i.e., cleavage C-terminal to Arg, not before Pro); one missed cleavage; and mass calibration and parameter optimization on. Fixed modifications included in the searches were as follows: carbamidomethylation of Cys (+57.02146) and TMT-pro labeling of Lys (+304.20715). Variable modifications were as follows: oxidation of Met (+15.9949); deamidation of Gln or Asn (+0.984016); acetylation of protein N-terminus (+42.0106); propionylation of protein N-terminus (+56.026215); peptide N-terminal TMT-pro labeling (+304.20715); peptide N-terminal pyroglu from Gln (−17.0265); peptide N-terminal pyroglu from Glu (−18.0106); peptide N-terminal methylation (+14.01565); peptide N-terminal dimethylation (+28.0313); peptide N-terminal trimethylation (+42.04695); peptide N-terminal formylation (+27.994915); peptide N-terminal myristoylation on Gly (+210.19836); and peptide N-terminal pyro-carbamidomethyl from carbamidomethyl Cys (−17.0265). A false discovery rate (FDR) threshold of <0.01 was applied for the PSMs. Following the initial search described above, data were searched again with a more limited set of variable modifications for quantitative differential expression analysis. This was due to the potentially negative effect that the increased search space and the very low detection rate of some N-terminal modifications might have on FDR threshold calculation. This limited set of modifications excluded the peptide N-terminal methylation, dimethylation, trimethylation, formylation, myristoylation, and pyro-carbamidomethyl. Furthermore, the search parameters for non-enriched samples were expanded to semi-specific ‘trypsin R’ digestion in order to permit the detection of neo-C-termini. The TMT quantitation of all samples was performed at the MS2 level, relative to a virtual reference channel derived from all channels in each TMTplex. An 8-core (16 Gb RAM) workstation was used for searches, which took between 50–90 min to complete, based on parameters used.

### 4.5. Statistical Analysis of TMT-Quantified Datasets

The normalized TMT reporter ion intensities were output from FragPipe; then, the significant differences between conditions were tested for via empirical Bayes moderated *t*-statistics within limma (v.3.54.1) [24] and Rstudio 4 (v.2202.07.02 build 576). Viral peptides were excluded from linear model fitting, and multiple test adjustments were performed using the Benjamini–Hochberg method (significance cutoff = 0.01; log2FC > 0.59).

### 4.6. Data Visualization

Data were visualized using in-house scripts and the following packages: ggseqlogo (v.0.1) for generated the sequence logos [25]; eulerr (v.7.0.0) for generating the Venn diagrams; and ggplot2 (v.3.4.1) for generating all other plots. The fragment spectra of the mass spectrometry data were visualized using PDV within FragPipe (FP-PDV-1.0.5) [26].

## Figures and Tables

**Table 1:** High-confidence 3CL^pro^ cleavage sites identified in PXD026797 pre-enrichment samples and not in N-termini-enriched samples. ↓, protease scissile bond.

UniProt Accession	Gene	Cleavage Site (P5–P5′)	Cleavage Site P1′ Position	Terminus Type	N-Terminal Label
P50454	SERPINH1	RSALQ↓SINEW	172	N-term	dimethyl
Q10567	AP1B1	SSKLQ↓SSNIF	879	N-term	dimethyl
Q13428	TCOF1	AAALQ↓AKSDE	1313	N-term	dimethyl
P11940	PABPC1	VAVLQ↓AHQAK	616	C-term	-
P26038	MSN	EALLQ↓ASRDQ	406	C-term	-
Q9UQ80	PA2G4	KALLQ↓SSASR	360	C-term	-
P36578	RPL4	EAQVQ↓ASVVK	363	C-term	-

## Data Availability

No new data were created or analyzed in this study. Data sharing is not applicable for this article.

## Supplementary Materials

The following supporting information can be downloaded at: https://www.mdpi.com/article/10.3390/ijms24108723/s1.
